# Development of Dietary Supplement Label Database in Italy: Focus of FoodEx2 Coding

**DOI:** 10.3390/nu12010089

**Published:** 2019-12-27

**Authors:** Alessandra Durazzo, Emanuela Camilli, Laura D’Addezio, Raffaela Piccinelli, Angelika Mantur-Vierendeel, Luisa Marletta, Paul Finglas, Aida Turrini, Stefania Sette

**Affiliations:** 1 CREA-Research Centre for Food and Nutrition, Via Ardeatina 546, 00178 Rome, Italy; emanuela.camilli@crea.gov.it (E.C.); laura.daddezio@crea.gov.it (L.D.); raffaela.piccinelli@crea.gov.it (R.P.); luisa.marletta@crea.gov.it (L.M.); aida.turrini@crea.gov.it (A.T.); stefania.sette@crea.gov.it (S.S.); 2 EuroFIR AISBL, 40 Rue Washington, 1050 Brussels, Belgium; am@eurofir.org; 3 Quadram Institute Bioscience, Norwich, Norfolk NR4 7UQ, UK; paul.finglas@quadram.ac.uk

**Keywords:** Dietary Supplement Label Database, dietary supplements, food description, food classification, FoodEx2

## Abstract

The sector of food supplements is certainly varied and growing: an ever wider offer of new products is launched on the market every year. This is reflected in new reorganization of drug companies and new marketing strategies, in the adoption of new production technologies with resulting changes in dietary supplements regulation. In this context, information on composition reported in labels of selected dietary supplements was collected and updated for the development of a Dietary Supplement Label Database according to products’ availability on the Italian market and also including items consumed in the last Italian Dietary Survey. For each item, a code was assigned following the food classification and description system FoodEx2, revision 2. A total of 558 products have been entered into the database at present, trying to give a uniform image and representation of the major classes of food supplements, and 82 descriptors have been compiled. Various suggestions on how the number of FoodEx2 system descriptors could be expanded were noted during the compilation of the database and the coding procedure, which are presented in this article. Limits encountered in compiling the database are represented by the changes in the formulation of products on the market and therefore by the need for a constant database update. The database here presented can be a useful tool in clinical trials, dietary plans, and pharmacological programs.

## 1. Introduction

The sector of food supplements is certainly varied and growing: a wider and wider selection of new products is launched on the market every year. This is reflected in new reorganization of drug companies, in new marketing strategies, and in the adoption of new production technologies with resulting changes in the dietary supplements regulation. The growth of this sector is encouraged by growing interest of consumers in improving their health and physical and mental wellbeing, often to compensate for an incorrect lifestyle [1].

Dietary supplements are considered in epidemiological studies and in the analysis of food consumption patterns [2]. There are several implications in dietary adequacy assessment especially with regard to the issue of upper limits in daily intake of certain nutrients. Moreover, several factors may influence the use of dietary supplements, such as gender, age, socio-economic status, educational level, dietary habits, etc. A first attempt to harmonize information on food supplements between European countries was performed by EFSA [3] with the purpose of producing a food composition database including both foods and food supplements to estimate nutrient intakes in European Countries. In this regard, it is worth mentioning some ongoing initiatives such as Global Dietary Database (GDD) (https://globaldietarydatabase.org/) and FAO/WHO Global Individual Food consumption data Tool (FAO/WHO GIFT) (http://www.fao.org/gift-individual-food-consumption/en/) aimed at the harmonization of dietary datasets worldwide for global diet monitoring using a common food classification and description system [4,5]. Considering the importance of dietary supplements in the evaluation of dietary intake, it is worth mentioning in particular the Dietary Supplement Label Database (DSLD) (https://dsld.nlm.nih.gov/dsld/) by the National Institutes of Health [6,7]; at present, it contains label information (brand name, ingredients, amount per serving, and manufacturer contact information) of more than 71,000 dietary supplements present and consumed in the U.S. marketplace [8,9]. The DSLD can be used to track changes in product composition and capture new products entering the market. Browsing options were developed and organized to search by product, ingredient, or contact of manufacturer, representing a unique resource that policymakers, researchers, clinicians, and consumers may find valuable for multiple applications [8,9].

In this context, information on composition reported on the product labels of selected dietary supplements has been collected and updated for the development of a Dietary Supplement Label Database for Italy, according to products’ availability on the Italian market and also including items from both the third Italian National Food Consumption Survey, INRAN-SCAI 2005-06 database [2] and the ongoing Italian national dietary survey IV SCAI. The design and construction of a food database requires above all identifying foods through an adequate food nomenclature and a precise description. The FoodEx2 system has been used for the classification and description of dietary supplements in the aforementioned database. FoodEx2 is a standardized food classification and description system developed by EFSA to better describe characteristics of foods and dietary supplements in exposure assessment studies; this system, nowadays at revised version 2, consists of flexible combinations of classifications and descriptions based on a hierarchical system for different food safety-related domains (i.e., food consumption, chemical contaminants, pesticide residues, zoonoses and food composition) [10,11,12,13,14]. This system is characterized by a compromise between comprehensiveness (sufficiently detailed description) and feasibility in different areas of food data collection. In fact, it consists of a fixed and sufficiently large set of food categories or groups (food classification—organization of terms identifying/assigning different food items into groups) defined at high level of detail that constitute the “core list” and represent the minimum recommended level for coding during data collection [15]. More detailed terms can be found on the “extended list”; terms present in the core and extended lists may be aggregated in a hierarchical parent–child relationship in several ways according to different food safety domains. Descriptors, defined “facets”, are aimed at registering all relevant food items characteristics and can be used to add details to create new categories responding to particular study requirements.

This work has been undertaken to study the application of FoodEx2 system starting from FoodEx2 categories (or terms) belonging to the FoodEx2 group “Products for non-standard diets, food imitates and food supplements” (A03RQ) for classifying the items that make up the Italian Dietary Supplement Label Database here presented.

## 2. Materials and Methods

The starting set of supplements has been drawn from the nationwide dietary surveys including the third Italian National Food Consumption Survey, INRAN-SCAI 2005-06 database [2] and items from preliminary results of the ongoing Italian national dietary survey IV SCAI. National food consumption surveys were designed with the aim of representativeness of the total population at national level and in the four main geographical areas, taking energy intake as the referring parameter.

Subsequently, products’ labels had been searched on the internet using the following keywords in Italian: dietary supplements, botanical, herbal formulations, vitamin-based supplements, mineral-based supplements, protein-based supplement, carnitine-based supplements, prebiotic formulations, probiotic formulations, algae-based formulations, enzyme-based formulations, yeast-based formulations, common supplements.

Afterwards, label surveys visiting retail points to directly observe products on shelves were carried out.

The official register of supplements authorized by the Italian Ministry of Health (http://www.salute.gov.it/imgs/C_17_pagineAree_3668_listaFile_itemName_1_file.pdf) was consulted.

The coding procedure was carried out by a qualified compiler who constantly follows the FoodEx2 system updates, taking part in training courses organized by system developers [14]. Another qualified compiler double-checked the codes.

Procedurally, information on composition of dietary supplements was taken from labels, and a code was assigned to each item following the food classification and description system FoodEx2, revision 2; the exposure hierarchy was used for coding [10,11,12,13,14]. The FoodEx2 categories (terms) belonging to the FoodEx2 group “Products for non-standard diets, food imitates and food supplements” (A03RQ) were considered for classification of the items.

FoodEx2 system consists of 21 clearly defined food groups. Detailed food groups represent the basis of the systems; a food only fits in one group and a parent–child structure is present within the food groups. Facets descriptors, of which there are 28 in total, can be viewed as characteristics of foods from different points of view; the facets give additional information for a peculiar aspect of food, i.e., part nature, ingredient, packaging material, production method, qualitative information, process, target consumer. Peculiarity of FoodEx2 is that each food group lists term with included implicit facet descriptors, to which further descriptors of different characteristics can be added; during compilation procedure, in FoodEx2 for each food item, the terms may be aggregated in different ways according to the needs [16]. “Implicit facets” means facets proper of the base term chosen for classification, and therefore, implicitly assigned to it, whereas “added facets” means the facet descriptors that are added by the coder to the chosen base term while coding a food item. The procedure consists of organizing them to reduce the coding time and prevent general imprecision.

For each food item, the terms may be aggregated in different ways according to the needs, without following a general scheme; a typical case is given by a base term, followed (optionally) by a hashtag “#” and a sequence of facet descriptors separated by dollar character “$”.

During this practical experience of compiling the Dietary Supplement Label Database, feedbacks and suggestions for possible enhancement of FoodEx2 were formulated and forwarded to system developers. These suggestions can be grouped as “Additional items”, “Clarifications”, and “Typing suggestions”.

## 3. Results and Discussion

A total of 558 products have been entered into the database at present, as an attempt to provide an adequate representation of the major categories of food supplements, and 82 descriptors have been compiled. Particular attention has been given to supplements/formulations based on medical herbs and plant extracts, one of the classes currently emerging [17,18].

### 3.1. Database Description

Items in the Dietary Supplements Label Database are organized in groups defined by base terms and additional facets.

Table S1 (Supplementary Material) reports ingredients and nutritional composition of the 558 products and Table S2 (Supplementary Material) reports the FoodEx2 codes of the 558 products.

#### 3.1.1. Base Terms

The base terms reported for describing the 558 products are distributed in the subgroups as follows: 73 Mixed supplements/formulations [A03TC], 28 Vitamin only supplements [A03SL], 27 Mineral only supplements [A03SM], 49 Combination of Vitamin and mineral only supplements [A03SN], 6 Bee-produced formulations [A03SQ], 7 Fiber supplements [A03SR], 283 Herbal formulations and plant extracts [A03SS], 14 Algae-based formulations (e.g., spirulina, chlorella) [A03ST], 8 Probiotic or prebiotic formulations [A0F3Y], 15 Formulations containing special fatty acids (e.g., Omega-3, essential fatty acids) [A03SX], 10 Protein and amino acids supplements [A03SY], 2 Coenzyme Q10 formulations [A03SZ], 1 Enzyme-based formulations [A03TA], 4 Yeast-based formulations [A03TB], 10 Other common supplements [A03SV], 3 Protein and protein components for sports people [A03SA], 6 Micronutrients supplement for sports people [A03SB], 7 Carnitine or creatine-based supplement for sports people [A03SC], 2 Nutritionally complete formulae [A03SE], 3 Imitation yoghurt, non-soy [A03TZ].

#### 3.1.2. Facets

Additional facets used for describing the dietary supplements are: FACET F03 “PHYSICAL STATE”, FACET F04 “INGREDIENT”, FACET F23 “TARGET CONSUMER”, FACET F33 “LEGISLATIVE CLASSES”. FACET F03 defines the physical state of a product such as: Tablets [A06JH], Powder [A06JD], Liquid [A06JL].

FACET F04 defines the characterizing ingredients. Common terms used are aggregation term Chemical elements [A0EVF], including core terms Calcium [A0EXH], Magnesium [A0EXF], Iron [A0EXD], Potassium [A0EXJ], Zinc [A0EXE], Fluorine [A0F3A]; aggregation term Vitamins [A0EVG], including core terms Vitamin C (Ascorbic acid) [A0EXN], Vitamin D (Cholecalciferol) [A0EXM], Vitamin E (Tocopherols, tocotrienols) [A0EXL, Vitamin A (retinol, carotenoids) [A0EXZ], Vitamin B9 (Folic acid, folinic acid) [A0EXQ], etc.; aggregation term Special fatty acids [A0EVS], including core terms Omega-3 fatty acids [A0EVV] and Omega-6 fatty acids [A0EVT]; aggregation term Phytochemicals [A0EVM], including core terms Phytosterols [A0EVQ], Polyphenols [A0EVP], Carotenoids [A0EVN]; the core term Dietary fiber [(A0EVR]; the extended terms Carnitine [A0F4N] and Creatine-creatinine [A0F4P]; the aggregation term Bee-produced fortifying agents [A0EVH], including the core term Royal jelly [A0CVG]; the aggregation term Live microorganisms for food production [A048X] including core terms Yeast cultures [A048Z]; the core term Caffeine [A0EVK]; the core term Algae-based fortifying agents (e.g., spirulina, chlorella) [A0EVL].

It is worth mentioning that the most used terms to indicate the ingredients present in the food supplements from the category Herbal formulations and plant extracts [A03SS] are Powdered extract of plant origin [A0ETZ], Liquid extract of plant origin [A0EVA], Extracts of plant origin [A0ETY], Dried herbs [A016T], Dried vegetables [A00ZQ], Dried fruit [A01MA], Dehydrated/powdered fruit juice [A03CG], Dehydrated/powdered vegetable juice [A03DA].

Examples of descriptors for FACET 23, used to indicate the target consumers for whom the product is intended are Children’s food [A07TL], including the Children’s food 4–8 years [A07TM] and Children’s food 9–15 years [A07TN]; Infant or toddler’s food [A07TF].

Within FACET F33, defining the legislative class, descriptors from the classification defined in the food additives legislation (Regulation (EC) No. 1333/2008) are used for dietary supplements as follows: FA-17.1 Food supplements supplied in a solid form including capsules and tablets and similar forms excluding chewable forms [A0C16], FA-17.2 Food supplements supplied in a liquid form [A0C15], FA-17.3 Food supplements supplied in a syrup-type or chewable form [A0C14].

#### 3.1.3. Groups’ Description

Examples of group Vitamin only supplements [A03SL] are dietary supplements containing vitamin D such as the product coded by [A03SL#F03.A06JH$F04.A0EXM$F33.A0C16] (Re-coded: Vitamin only supplements, STATE = Tablets, INGRED = Vitamin D (cholecalciferol), LEGIS = FA-17.1 Food supplements supplied in a solid form including capsules and tablets and similar forms, excluding chewable forms) or the one by [A03SL#F03.A06JL$F04.A0EXM$F33.A0C15] (Re-coded: Vitamin only supplements, STATE = Liquid, INGRED = Vitamin D (cholecalciferol), LEGIS = FA-17.2 Food supplements supplied in a liquid form); dietary supplements containing vitamin C such as the product coded by [A03SL#F04.A0EXN$F33.A0C14] (Re-coded: Vitamin only supplements, INGRED = Vitamin C (ascorbic acid), LEGIS = FA-17.3 Food supplements supplied in a syrup-type or chewable form); dietary supplements containing vitamin B9 such as the product coded by [A03SL#F03.A06JH$F04.A0EXQ$F33.A0C16], (Re-coded: Vitamin only supplements, STATE = Tablets, INGRED = Vitamin B9 (folic acid, folinic acid), LEGIS = FA-17.1 Food supplements supplied in a solid form including capsules and tablets and similar forms, excluding chewable forms); etc.

For group Mineral only supplements [A03SM], including all supplements based only on minerals, examples are dietary supplements containing, i.e., iron, potassium, magnesium, zinc, or combination such as potassium and magnesium widespread used. An example of FoodEx2 code of a product containing potassium and magnesium is as follows: FoodEx2 code [A03SM#F03.A06JH$F04.A0EXJ$F04.A0EXF$F33.A0C16], Re-coded: Mineral only supplements, STATE = Tablets, INGRED = Potassium, INGRED = Magnesium, LEGIS = FA-17.1 Food supplements supplied in a solid form including capsules and tablets and similar forms, excluding chewable forms.

Examples for group Combination of vitamin and mineral only supplements [A03SN] that comprises all supplements based only on formulations including both minerals and vitamins are dietary supplements containing vitamin D and Calcium, i.e., FoodEx2 Code: [A03SN#F04.A0EXM$F04.A0EXH], Re-coded: Combination of vitamin and mineral only supplements, INGRED = Vitamin D (cholecalciferol), INGRED = Calcium; dietary supplements containing vitamin C and iron, i.e., FoodEx2 Code: [A03SN#F03.A06JH$F04.A0EXN$F04.A0EXD$F33.A0C16], Re-coded: Combination of vitamin and mineral only supplements, STATE = Tablets, INGRED = Vitamin C (ascorbic acid), INGRED = Iron, LEGIS = FA-17.1 Food supplements supplied in a solid form including capsules and tablets and similar forms, excluding chewable forms.

The Herbal formulations and plant extracts [A03SS] include any type of supplement based on herbal formulations and/or plant extracts. Typical ingredients are ginkgo biloba, dog rose, star anise, tamarind, aloe, rhubarb, acacia, dandelion, astragalus, psyllium, holy basil, sage and others; these occur as dried products or liquid or powdered extracts. In addition to the classic medical herbs just mentioned, there are also foods with functional components such as artichoke, garlic, pineapple, black currant, whose use has become frequent. An example of coding of artichoke- based product is FoodEx2 Code: [A03SS#F03.A06JL$F04.A0EVA$F33.A0C15], (Re-coded: Herbal formulations and plant extracts, STATE = Liquid, INGRED = Liquid extract of plant origin, LEGIS = FA-17.2 Food supplements supplied in a liquid form), and in the Remark is noted: “The ingredient indicated as Liquid extract of plant origin is hydroalcoholic extract of artichoke leaves”. For a garlic-based product, FoodEx2 Code is [A03SS#F04.A0ETZ$F33.A0C16] (Re-coded: Herbal formulations and plant extracts, INGRED = Powdered extract of plant origin, LEGIS = FA-17.1 Food supplements supplied in a solid form including capsules and tablets and similar forms, excluding chewable forms) and in the Remark is noted: “The ingredient indicated as Powdered extract of plant origin is garlic bulb dry extract”.

The group Other Common Supplements [A03SV], referring to any type of other common supplements, includes formulations containing, as the main ingredient, compounds such as alpha lipoic acid, beta glucans, lactoferrin, melatonin, etc. An example of product containing lipoic acid is given by the code [A03SV#F03.A06JH$F04.A0F4M$F33.A0C16], (Re-coded: Other common supplements, STATE = Tablets, INGRED = Co-factors to metabolism, LEGIS = FA-17.1 Food supplements supplied in a solid form including capsules and tablets and similar forms, excluding chewable forms) and in the Remark is noted “The ingredient indicated as Co-factors of metabolism is α-lipoic acid”.

Another example is given by a product containing melatonin coded as [A03SV#F03.A06JH$F04.A0EVM$F33.A0C16] (Re-coded: Other common supplements, STATE = Tablets, INGRED = Phytochemicals, LEGIS = FA-17.1 Food supplements supplied in a solid form including capsules and tablets and similar forms, excluding chewable forms) and in the Remark is noted “The ingredient indicated as phytochemicals is melatonin”.

The group Mixed supplements/formulations [A03TC] includes any type of supplements combining different principles without a strong prevalence of one. Moreover, various products belonging to this group present bioactive molecules among the ingredients, such as rutin, quercetin, coenzyme Q10. An example is given by product containing powdered extract of plant origin and fish oil, quercetin, vitamin C, vitamin B5, and Methylsulfonylmethane; it is coded as [A03TC#F03.A06JH$F04.A0ETZ$F04.A0EVP$F04.A0EXN$F04.A0EXT$F04.A038M$F04.A0EVV$F33.A0C16] (Re-coded by Mixed supplements/formulations, STATE = Tablets, INGRED = Powdered extract of plant origin, INGRED = Polyphenols, INGRED = Vitamin C (ascorbic acid), INGRED = Vitamin B5 (pantothenic acid), INGRED = Fish oil, INGRED = Omega-3 fatty acids, LEGIS = FA-17.1 Food supplements supplied in a solid form including capsules and tablets and similar forms, excluding chewable forms) and in the Remark is noted “The ingredients indicated as licorice root dry extract, plantain leaves dry extract, chamomile flowers dry extracts, nettle aerial parts. The ingredient indicated as Polyphenols is quercetin. Methylsulfonylmethane is also contained”.

### 3.2. Feedback and Suggestions for FoodEx2 Revision 2 Implementation: Focus on Dietary Supplements

Here we report the feedback and suggestions for implementation of FoodEx2 formulated during the development and updating of Dietary Supplement Label Database in Italy. Concerning additional items, supplementary aggregation terms for proteins and amino acids should be added, including core terms for main amino acids used in dietary supplements as well as within “Special fatty acids”, extended terms for “Omega-3 fatty acids” and “Omega-6 fatty acids”. Considering the widespread growth in the consumption of herbal remedies, additional items, such as powdered dried fruit, powdered dried vegetables and powdered dried herbs would be very useful. At the same time, attention should be given to additional terms linked to description of bioactive compounds; in this order, within the aggregation term “Phytochemicals”, several core terms, i.e., alkaloids, nitrogen-containing compounds, organosulfur compounds, should be added, including their corresponding extended terms. Moreover, extended terms should be associated to the core terms just present in FoodEx2 System, “Carotenoids” and “Polyphenols”.

In line with technological progress, facet descriptors, i.e., capsules, softgels, opercula, chewable tablets, and gastro-resistant tablets should be considered.

Details on additional items proposed were reported in Table 1.

“Clarification” about “scope notes”(textual information helping describing the selected term) of “protein and amino acids supplements” and “protein and protein components for sports people” should be underlined; differences in “protein and amino acids supplements [A03SY]” and “protein and protein components for sports people [A03SA]” should be clarified as well as if dietary supplements containing fiber with a marked prebiotic activity should be included in “Fiber supplements [A03SR]” or “Probiotic or prebiotic formulations [A0F3Y]”. Moreover, several typing suggestions were indicated, i.e., “Chemical elements” should be replaced by “Minerals” and “Fiber” by “Fibre”.

## 4. Conclusions

A total of 558 products have been entered into the database at present, with the aim of providing an adequate representation of the major classes of food supplements, and 82 descriptors have been compiled.

This paper represents one of first works describing the procedure of coding dietary supplements through the FoodEx2 classification system and could be a useful tool/guide for other compilers and users.

The Dietary Supplement Label Database here presented is intended to be a first example of building a database of information on marketed dietary supplements and provides several suggestions for improving the adopted classification coding system. This database is intended as a basis for a dynamic database that can be expanded as new products are offered on the market. The main feature of a database dedicated to food supplements is its intrinsic dynamism linked to the frequent changes in the formulation of food supplements, with the consequent need to monitor the market and update the database regularly, both by inserting new formulations and expanding the number of descriptors. A precise and available description of the dietary supplements through coding is essential to recognize the type, the main ingredients, and the target consumers by users from different countries.

This database will help consumers to make healthy choices and will represent a valid tool for dietary intake calculations. This database can be useful in different contexts, such as, for example, in clinical trials, dietary plans and pharmacological programs, but also to expand the food composition databases for the purpose of daily nutrient intake estimations.

Considering the integrity of the labels of dietary supplements and whether they reflect the actual amount of each ingredient contained in the product or not, as properly pointed by Betz et al. [19], a new challenge is given by the development of analytically validated laboratory-derived dietary supplement databases. A valid, rapid, and environmental friendly tool in this direction could be represented by the use of infrared spectroscopy joined with chemometrics in the perspective of integrated research approach; as for instance, the development of a “fingerprint spectra database” of dietary supplements could be useful for further researches and applications in the assessment of quality and safety, i.e., monitoring production and/or shelf life of a product, identifying contaminants, and confirming an incoming product.

## Figures and Tables

**Table 1:** Proposed descriptors for implementation of FoodEx2 distinguished by type of term in FoodEx2 hierarchical structure *.

Aggregation Terms	Core Terms	Extended Terms
**AMINO ACIDS**	**ARGININE**	
	**ASPARTIC ACID**	
	**GLUTAMINE**	
	**VALINE**	
	**GLYCINE**	
	**LEUCINE**	
	**ISOLEUCINE**	
	**LYSINE**	
	**METHIONINE**	
	**THREONINE**	
	**TRYPTOPHAN**	
	**TYROSINE**	
	**BETA-ALANINE**	
	**PHENYLALANINE**	
	**CYSTEINE**	
	**HISTIDINE**	
**PROTEINS**		
Chemical elements [A0EVF]	**SODIUM**	
	**CHLORINE**	
	**BORON**	
	**CHROME**	
	**TIN**	
	**NICKEL**	
	**SILICON**	
Special fatty acids [A0EVS]	Omega-3 fatty acids [A0EVV]	**DOCOSAHEXAENOIC ACID (DHA)**
		**EICOSAPENTAENOIC ACID (EPA)**
		**ALPHA LINOLENIC ACID (ALA)**
	Omega-6 fatty acids [A0EVT]	**LINOLEIC ACID**
		**ARACHIDONIC ACID**
		**GAMMA LINOLENIC ACID (GLA)**
	**OLEIC ACID**	
Phytochemicals [A0EVM]	Carotenoids [A0EVN]	**BETA-CAROTENE**
		**LUTEIN**
		**ASTAXANTHIN**
	Polyphenols [A0EVP]	**PHENOLIC ACIDS**
		**FLAVONOIDS**
		**STILBENES**
		**LIGNANS**
	**ALKALOIDS**	
	**ORGANOSULFUR COMPOUNDS**	
	**NITROGEN-CONTAINING COMPOUNDS**	
Other plant oils [A037L]	**BORAGE OIL**	
	**ROSEMARY OIL**	
	**EVENING PRIMROSE OIL**	
	Brown algae [A00VK]	**FUCUS**
		**ASCOPHYLLUM NODOSUM**
	Green algae [A00VB]	**CHLORELLA**
	Dried fruit [A01MA]	**POWDERED DRIED FRUIT**
	Dried vegetables [A00ZQ]	**POWDERED DRIED VEGETABLES**
	Dried herbs [A016T]	**POWDERED DRIED HERBS**

* Proposed FoodEx2 descriptors are in upper case and bold.

## Supplementary Materials

The following are available online at https://www.mdpi.com/2072-6643/12/1/89/s1, Table S1: Ingredients and nutritional composition of the 558 products; Table S2: FoodEx2 codes of the 558 products.
